# Supplementary material for: Latitude-dependent oxygen fugacity in arc magmas
Source: Nat Commun. 2024 Jul 18;15:6050. doi: 10.1038/s41467-024-50337-6 (PMC11258285; doi:10.1038/s41467-024-50337-6)
Supplement: Supplementary file 1 — Supplementary Information [file 41467_2024_50337_MOESM1_ESM.pdf]

Supplementary Information for

Latitude-dependent oxygen fugacity in arc magmas

Fangyang Hu[1], Hehe Jiang[1], Bo Wan[1], Mihai N. Ducea[2,3], Lei Gao[4], Fu-Yuan Wu[1,5]

[1]State Key Laboratory of Lithospheric and Environmental Coevolution, Institute of Geology and Geophysics, Chinese Academy of Sciences, Beijing, China

[2]Faculty of Geology and Geophysics, University of Bucharest, Bucharest, Romania

[3]Department of Geosciences, University of Arizona, Tucson, AZ, USA

[4]State Key Laboratory of Geological Processes and Mineral Resources, School of Earth Sciences and Resources, China University of Geosciences, Beijing, China

[5]College of Earth and Planetary Sciences, University of Chinese Academy of Sciences, Beijing, China

This file includes:

Supplementary Methods

Supplementary Figures 1-14

Supplementary Methods

Data compilation and selection

Data selection and filtration are fundamental for subsequent data interpretation. We chose circum-pacific arcs as our studied objects because the paleo-latitudes of these arcs remained basically unchanged during the Cenozoic[1]. Such a compilation can also provide much more data than only selected data of arc basaltic rocks formed during the Quaternary.

The V/Sc ratio is selected to reflect the oxygen fugacity in consideration of its widespread use and relatively insensitive to low-temperature alteration, metamorphism, and the effects of differentiation and degassing for high-MgO primary basalts[1–6]. The presence of garnet in the mantle source will influence the V/Sc ratio of magma, resulting in higher V/Sc ratios than those without garnet in the source at the same oxygen fugacity[4]. Turner and Langmuir[7] suggested that the garnet is present for continental arcs with thick crust. However, our compiled data showed that the V and Sc have no positive relationship with Dy/Yb, which are all compatible in garnet (Supplementary Fig. 2a). In addition, V and Sc show similar behavior of decreasing with Dy/Yb ratios increasing, indicating that the crustal thickness may have little influence on the V/Sc ratios (Supplementary Fig. 2a). As shown in Supplementary Fig. 2, the V/Sc ratios have no clear correlations with La/Yb, Dy/Yb ratios, and Moho depths, contrasting with a linear relationship between Dy/Yb and Moho depths, indicating that our compiled data of V/Sc ratios are not influenced by the effect of garnet melting. The crustal thickness is mainly related to the extent of melting[7–9], which can be evaluated by geochemical modeling. The Cu/Zr ratio is selected because it is a recently proposed indicator of oxygen fugacity of mantle and represents a chalcophile element melting behavior[8] that provides independent information of oxygen fugacity from the lithophile V/Sc systematics. As shown in Supplementary Fig. 2, the Cu/Zr ratio is more sensitive to the melting degree and crustal thickness than V/Sc ratio, which is consistent with previous evaluations[8]. Hence, the Cu/Zr ratios cannot directly linked to the oxygen fugacity of mantle.

The V/Sc and Cu/Zr ratios of magmatic rocks will increase or decrease during magma differentiation processes[4,8]. Therefore, it is crucial to obtain the data of primary basaltic rocks, which could reflect the oxygen fugacity of mantle. Previous studies have employed several criteria. For example, Lee et al.[4] pointed out that MgO>wt.8% is required because the clinopyroxene mainly crystallizes when MgO<8wt.%, which will greatly influence the V/Sc ratio of magma. Mallmann

and O'Neill[10] chose the MgO>6wt.%, and Stolper and Bucholz[2] chose the samples with MgO>6.5wt.% to minimize the effect of crystallization of arc magmas. In addition, Stolper and Bucholz[2] chose the samples with MgO=8-15wt.% for mid-ocean ridge basalt. Wang et al.[6] proposed that samples with SiO_2<52wt.%, MgO>8wt.%, Mg#=60-72, and Ni>100ppm are selected. In addition, Zhao et al.[8] suggested that only a small part of arc samples having MgO>8wt.%, and therefore, they proposed that samples with MgO>6.0wt.%, and Mg#>60 represent primitive magma compositions after evaluations. Wang et al.[6] excluded the alkaline magmas, boninites, and high-Mg andesites, and therefore only arc basaltic rocks are selected. The alkaline volcanic rocks (mostly basanites, and trachybasalts) in arc setting are mainly formed by partial melting of mantle wedge which is significantly modified by recycled oceanic arc crust components[11–13]. The geochemical compositions of these rocks resemble the oceanic island basalt (OIB), with different geochemical signatures with the typical arc basalts, such as high Ti and Nb contents and Ce/Y and V/Sc ratios[11,14]. The boninites are formed during the initial subduction, and the mantle wedge has not been sufficiently modified by the slab-derived materials[15–18]. Therefore, the boninites mainly reflects the oxygen fugacity of mantle wedge prior to subduction. The high-Mg andesites are proposed to be differentiated from primary arc andesite magmas produced by the reaction of adakites with the overlying mantle wedge[19]. The adakites are mainly formed by the partial melting of subducted slab[20], which will also not reflect the composition of the mantle wedge. In consideration of all these criteria and our compiled data (Supplementary Figs. 11-14), we then selected samples in both arcs and mid-ocean ridges with SiO_2=45-52wt.%, MgO=6.5-15wt.%, and Mg#=60-72. The alkaline magmas are excluded by using the Rittmann index[21] ($\sigma=(Na_2O+K_2O)^2/(SiO_2-43)$), and the samples with σ higher than 3.5 are alkaline. The boninites, high-Mg andesites, and adakites are also excluded.

The selected samples for different arc segments are calculated for the average values and standard error following the method applied by Stolper and Bucholz[2]. The arc volcanos from central part of the Vanuatu arc are influenced by the collision with the d'Entrecasteaux ridge and show clear differences with the northern and southern arc segments[22]. Therefore, the samples from the central part of the arc may not be suitable for compilation. Some samples from the Central American Arc have distinctly high Nb contents and low Sc contents, which is proposed to be related to the subduction of seamounts[23,24]. Therefore, these samples are also excluded from the dataset.

The data from the South Sandwich arc show very low Ti contents with high Sc contents,

implying a quite high melting degree (Supplementary Fig. 1). However, both V/Sc and Cu/Zr proxies indicate a relatively low redox state compared to other arc segments (Fig. 4 and Supplementary Figs. 5 and 6). It is noteworthy that the volcanic rocks from the South Sandwich arc have abnormally high $\delta^{11}B$ values (~15‰) with low B/Nb ratios, which is clearly different with other typical arcs and could be caused by subduction erosion of fore-arc peridotite[25,26]. Therefore, the South Sandwich is a rare case, and the source may be unique compared to other arc segments[27] and we exclude it from regressions but display the data on diagrams. The basaltic rock of South Shetland is still under debated as to whether they are subduction related[28,29]. As a result, we kept them on the diagram and included for the regression but leaving with a dotted line. The data from the Luzon arc is quite dispersive with high standard deviation values, and therefore, we also exclude them from regressions. Finally, the outliers are excluded based on the analysis of the modified Thompson-tau method.

Uncertainty of fO_2 calculated by V/Sc-Ti system

The uncertainty of fO_2 calculated by V/Sc-Ti system has been evaluated by Gao et al.[30] and Wang et al.[6], including the (1) analytical uncertainties of trace elements(<5%, RSD), (2) the uncertainties of the initial mantle wedge composition (7% for V, 13% for Sc, and 12% for Ti)[31], (3) uncertainties of P-T estimations, and (4) systematic biases in the functions. The uncertainty (3) and (4) will directly result in the uncertainty of the partition coefficients by using the equations proposed by Wang et al.[6]. The analytical uncertainties of trace elements by ICP-MS are typically better than 5% (1 RSD), which could cause the uncertainty of calculated ΔFMQ of ~0.1-0.2 log unit (1 SD). The uncertainty of the initial mantle compositions is evaluated in Supplementary Fig. 5, which results in the uncertainty of ~0.1 log unit as well. According to the empirical equations obtained by Wang et al.[6], the partition coefficients of minerals are functions of melting temperature and pressure, oxygen fugacity, and mineral and melt compositions. Based on our calculation of the compiled data, the uncertainty of temperature and pressure are 33°C and 0.24GPa, respectively, and the uncertainty of NBO/T of magma is 0.14 (Supplementary Data 2). The mineral compositions are set to be constant. The coefficients in functions are also set to be constant because these numbers are directly related to the experimental data used for regressions, which is independent of influence factors of partial melting processes. Therefore, according to the empirical equations and mineral modes during

partial melting, the propagated uncertainty of bulk partition coefficient of V and Sc are about 0.06 and 0.04, respectively. As a result, the uncertainty of calculated V/Sc ratios based on the partial melting model is approximately equal to 0.8-1.0 (1 SD), which in turn leads to a ~0.2-0.3 log unit (1 SD) uncertainty of the calculated ΔFMQ. Therefore, the bulk uncertainty of calculated ΔFMQ is proposed to be about 0.4-0.5 log unit (1 SD), consistent with previous estimations[30,32,33].

Since this method is purely model-based, we suggest that the difference in the modeled fO_2 based on V/Sc variations across latitudes is more significant than the absolute values of modeled fO_2. Therefore, although the uncertainty is quite large for our estimation of the difference of ΔFMQ between the high latitude and low latitude arcs, it will not influence our interpretation of the latitudinal variations of V/Sc ratios. For instance, because the V, Sc, and Ti are all incompatible elements, the variations of these elements in the mantle will increase or decrease simultaneously, resulting in the calculated fO_2 isopleths changing as a whole (Supplementary Fig. 5). In addition, the NBO/T of melt, melting pressure, and temperature have no clear correlations with latitudes. Thus, the uncertainties of these factors will not lead to the partition coefficients varying with the latitudes. During the modeling, all the coefficients in the functions are set as constants, and therefore, the calculated fO_2 isopleths will also be changed as a whole. In this way, the V/Sc-inferred ΔFMQ values could be changed when different parameters are applied, but the inferred ΔFMQ values of high V/Sc ratios will always be higher than those of low V/Sc ratios. Only analytical errors may influence our interpretations because such uncertainty is directly related to our observations. However, the analytical uncertainties have a limited effect on the calculated ΔFMQ values. Therefore, we suggest that the uncertainty of this method will not influence our interpretation of the inferred variations of oxygen fugacity based on V/Sc ratios.

Supplementary Figures

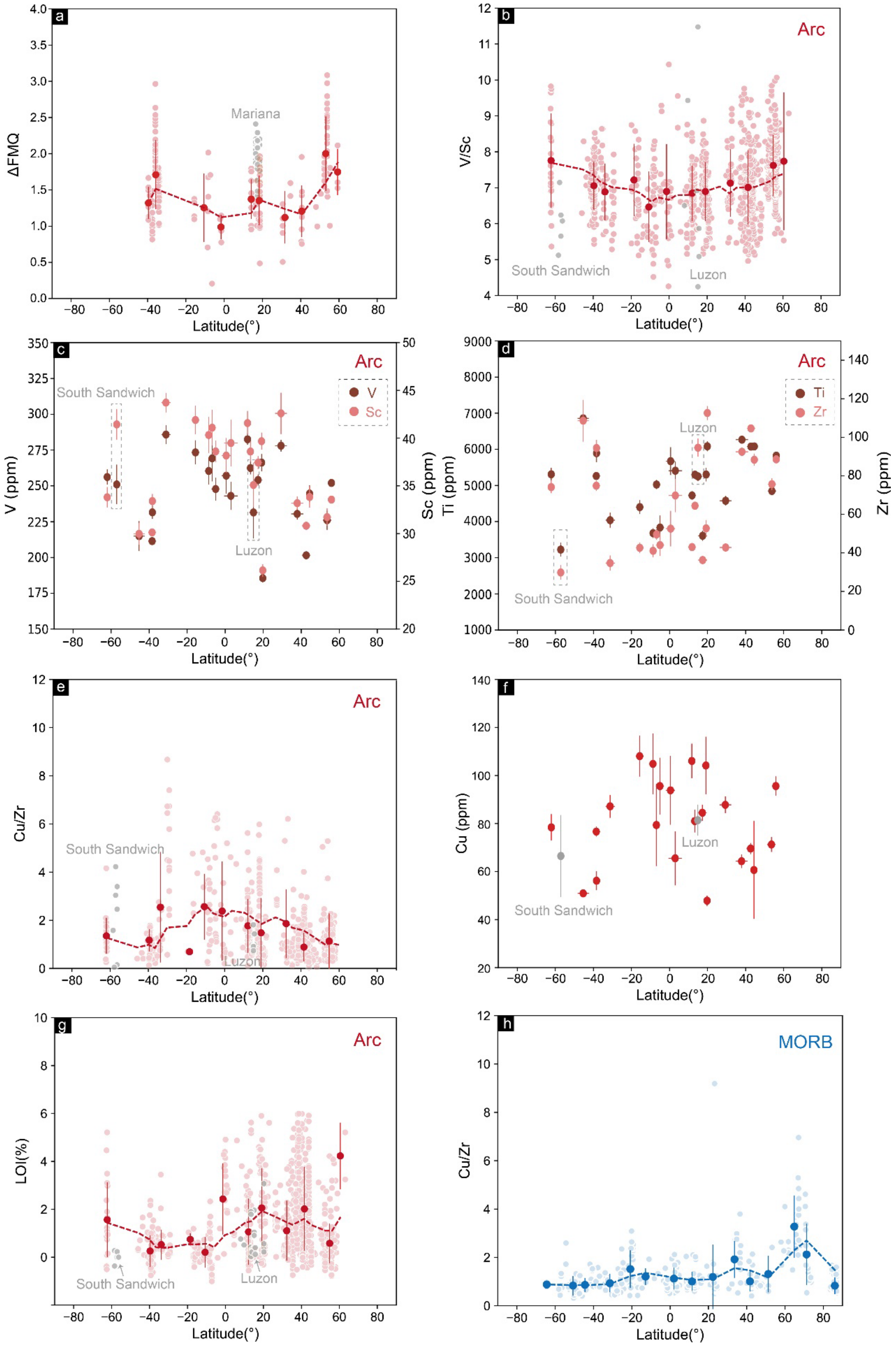

Supplementary Figure 1. Compiled whole-rock data of primary arc basalts and mid-ocean ridge basalts (MORB). (a) Scatter plot of measured/calculated ΔFMQ of olivine-hosted melt inclusions

from arc basaltic rocks versus latitude (°) with average values and standard deviations (box size of 10°). The calculated moving average curve is also plotted. The data in gray are ΔFMQ values of Mariana arc calculated based on olivine-melt V partitioning using the data presented in Brounce[34]. (b) Scatter plot of V/Sc ratios of arc basaltic rocks versus latitude (°) with average values and standard deviations (box size of 10°). The calculated moving average curve is also plotted. (c) Scatter plot of average V (ppm) and Sc (ppm) contents of basaltic rocks from different arc segments versus latitude (°). The error bar is 1SE. (d) Scatter plot of average Ti (ppm) and Zr (ppm) contents of basaltic rocks from different arc segments versus latitude (°). The error bar is 1SE. (e) Scatter plot of Cu/Zr ratios of arc basaltic rocks versus latitude (°) with average values and standard deviations (box size 10°). The calculated moving average curve is also plotted. (f) Scatter plot of average Cu (ppm) contents of basaltic rocks from different arc segments versus latitude (°). The error bar is 1SE. (g) Scatter plot of loss on ignition (LOI) of arc basaltic rocks versus latitude (°) with average values and standard deviations (box size of 10°). The calculated moving average curve is also plotted. (h) Scatter plot of Cu/Zr ratios of MORB versus latitude (°) with average values and standard deviations (box size 10°). The calculated moving average curve is also plotted. The compiled data are listed in Supplementary Data 1, 2, 4, and 5.

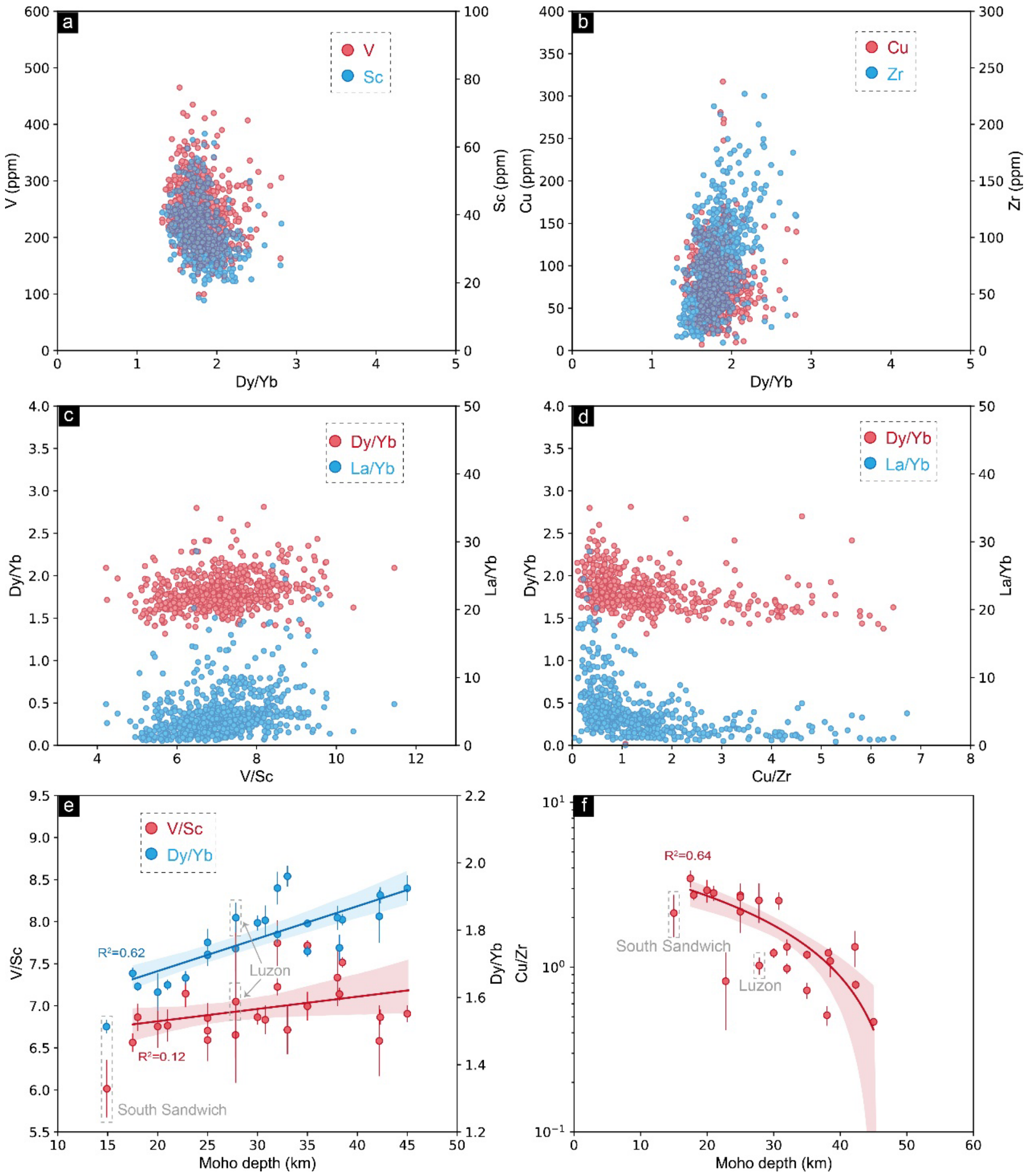

Supplementary Figure 2. Influences of crustal thickness on compiled data of arc basaltic rocks. (a) V (ppm) and Sc (ppm) versus Dy/Yb. (b) Cu (ppm) and Zr (ppm) versus Dy/Yb. (c) Dy/Yb and La/Yb versus V/Sc. (d) Dy/Yb and La/Yb versus Cu/Zr. (e) V/Sc and Dy/Yb versus Moho depth (km). The error bar is 1SE. The data from South Sandwich and Luzon are excluded for regressions. (f) Cu/Zr versus Moho depth (km). The error bar is 1SE. The data from South Sandwich and Luzon are excluded for regressions. The compiled data are listed in Supplementary Data 2 and 5.

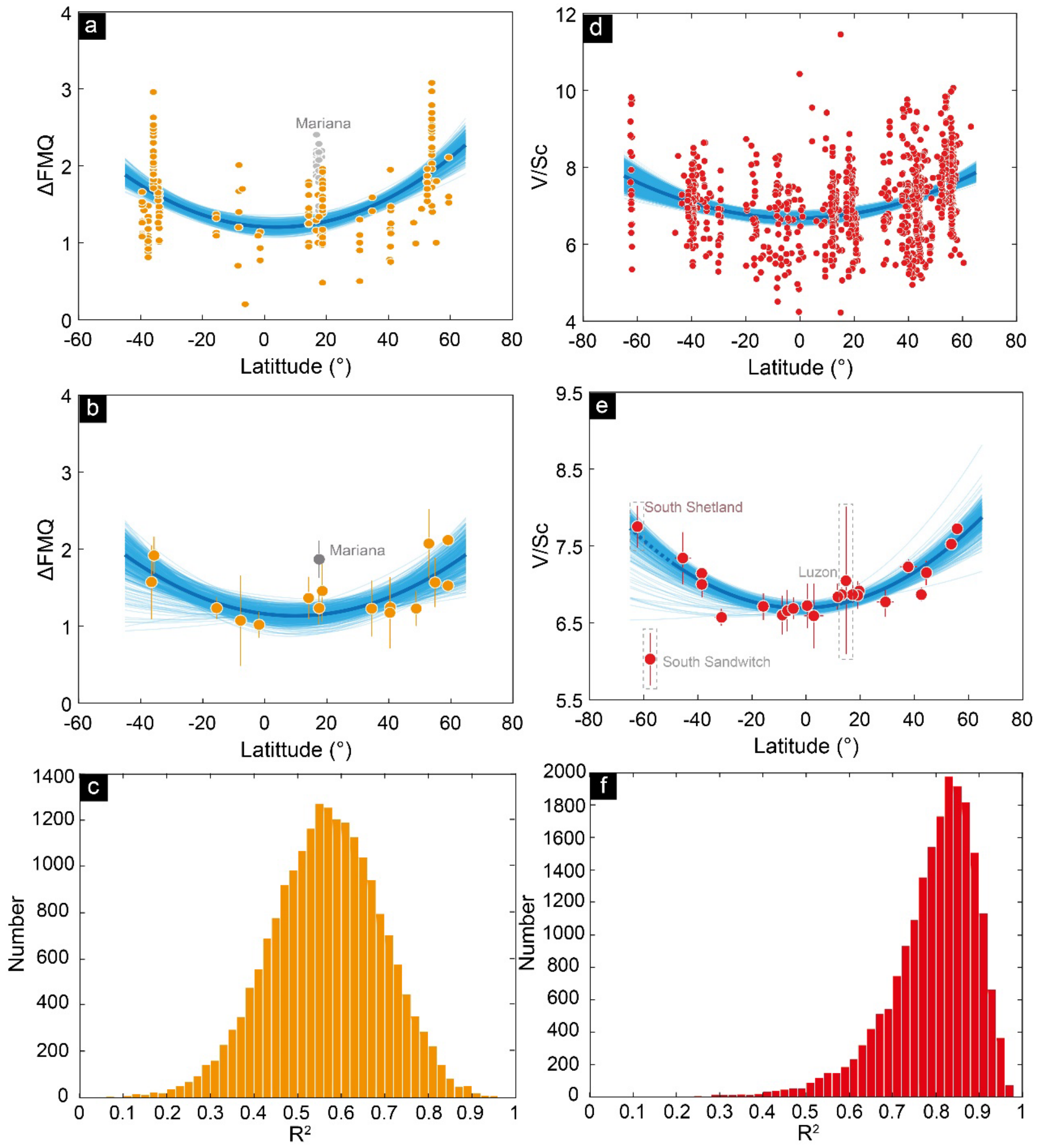

Supplementary Figure 3. Relationships between the ΔFMQ of olivine-hosted inclusions from arc basaltic rocks and V/Sc of arc basaltic rocks and latitude. Thin blue lines are bootstrap Monte Carlo resampling results and thick blue lines are the mean of 20000 calculated polynomial regressions. (a) Scatter plot of measured ΔFMQ of olivine-hosted melt inclusions from arc basaltic rocks versus latitude. The data in gray are ΔFMQ values of Mariana arc calculated based on olivine-melt V partitioning using the data presented in Brounce[34], which are not included for regression. (b) Scatter plot of average ΔFMQ of olivine-hosted melt inclusions from arc basaltic rocks versus latitude. The data from Luzon is excluded. The Mariana represents data of ΔFMQ values calculated based on olivine-melt V partitioning presented in Brounce[34]. The error bar is 1SD. (c) Histogram of R^2 values of regressions plotted in Supplementary Fig. 3b. (d) Scatter plot of V/Sc ratios of arc basaltic rocks versus latitude. (e) Scatter plot of average V/Sc ratios of arc basaltic rocks versus latitude. The data from South Sandwich is excluded from analysis. The error bar is 1SE. (f) Histogram of R^2 values of regressions plotted in Supplementary Fig. 3e.

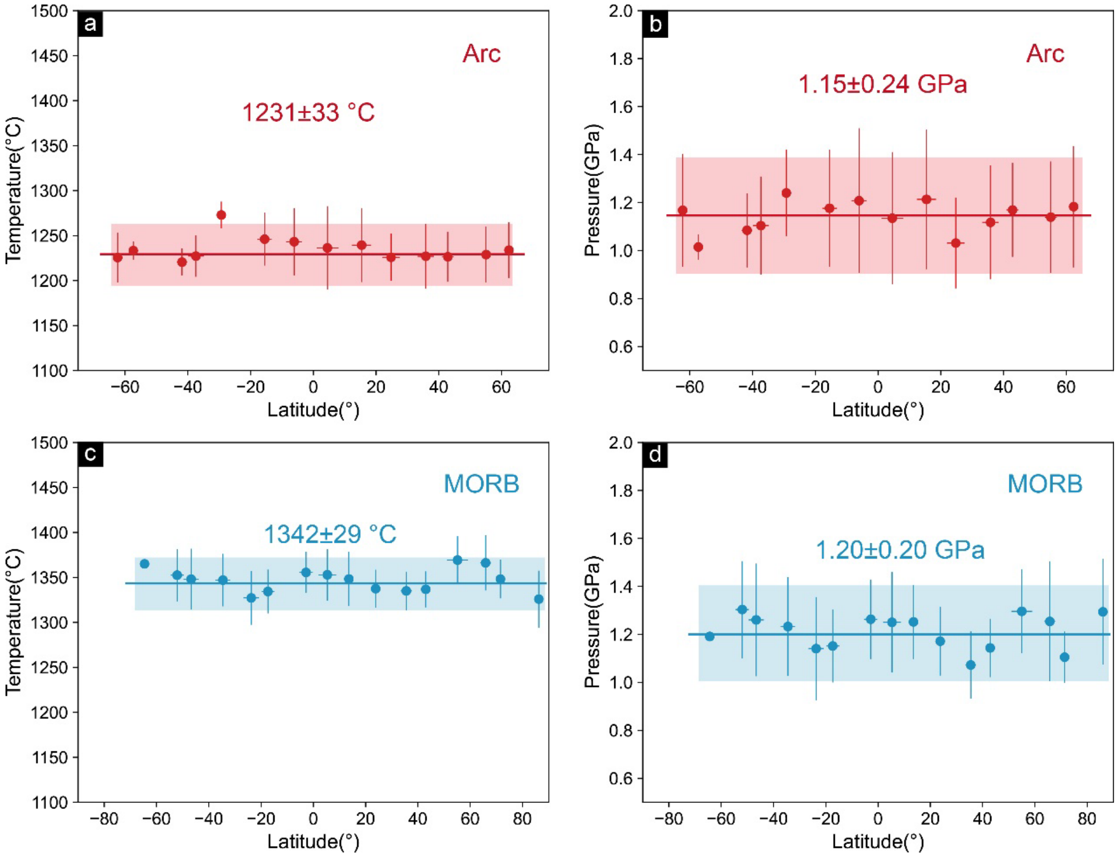

Supplementary Figure 4. Variations of calculated melting temperature and pressure of primary arc basalts (a, b) and mid-ocean ridge basalts (MORB) (c, d) along the latitude. The calculation method is based on the Lee et al.[35]. Please see Methods for parameters used for calculations. The calculated average values and standard deviations are plotted with a bin size of 10°. The average values and standard deviations of all calculated data are also shown. The calculated data are shown in Supplementary Data 2 and 4.

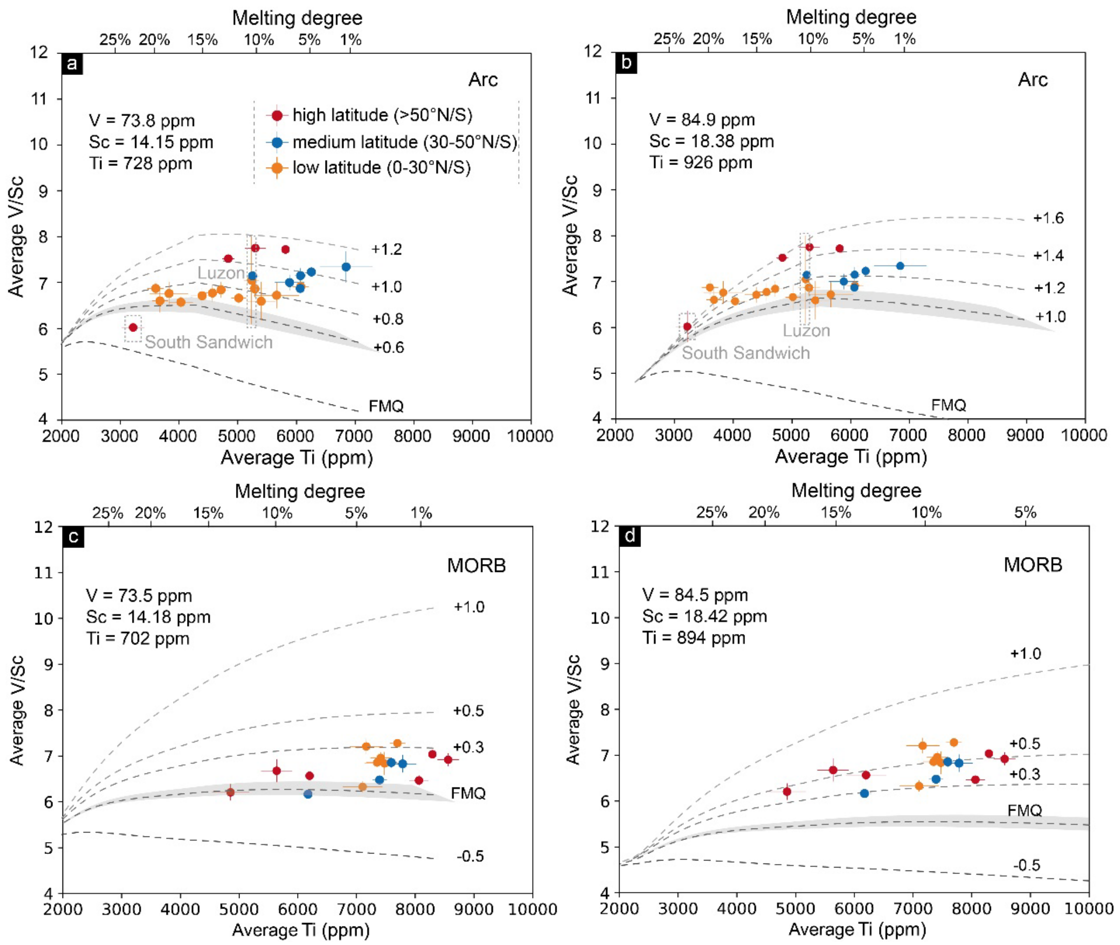

Supplementary Figure 5. Estimated oxygen fugacity of arc basaltic rocks and MORB based on V/Sc versus Ti (ppm). (a, b) The fO_2 estimation for arc basaltic rocks is modeled at V=73.8ppm or 84.9ppm, with Sc=14.15ppm or 18.38ppm and Ti=728ppm or 926ppm. (c, d) The fO_2 estimation for MORB is modeled at V=73.5ppm or 84.5ppm, with Sc=14.18ppm or 18.42ppm and Ti=702ppm or 894ppm, illustrating influence of source variations on oxygen fugacity estimation. The calculated fO_2 isopleths are modeled at melting temperature of 1231±33°C and melting pressure of 1.15±0.24 GPa for arc basaltic rocks and melting temperature of 1342±29°C and melting pressure of 1.20±0.20 GPa for MORB (Supplementary Fig. 3). The shaded regions represent the range of estimated melting pressures and temperatures. The modeling results are shown in Supplementary Data 7 and 8. The error bar is 1SE.

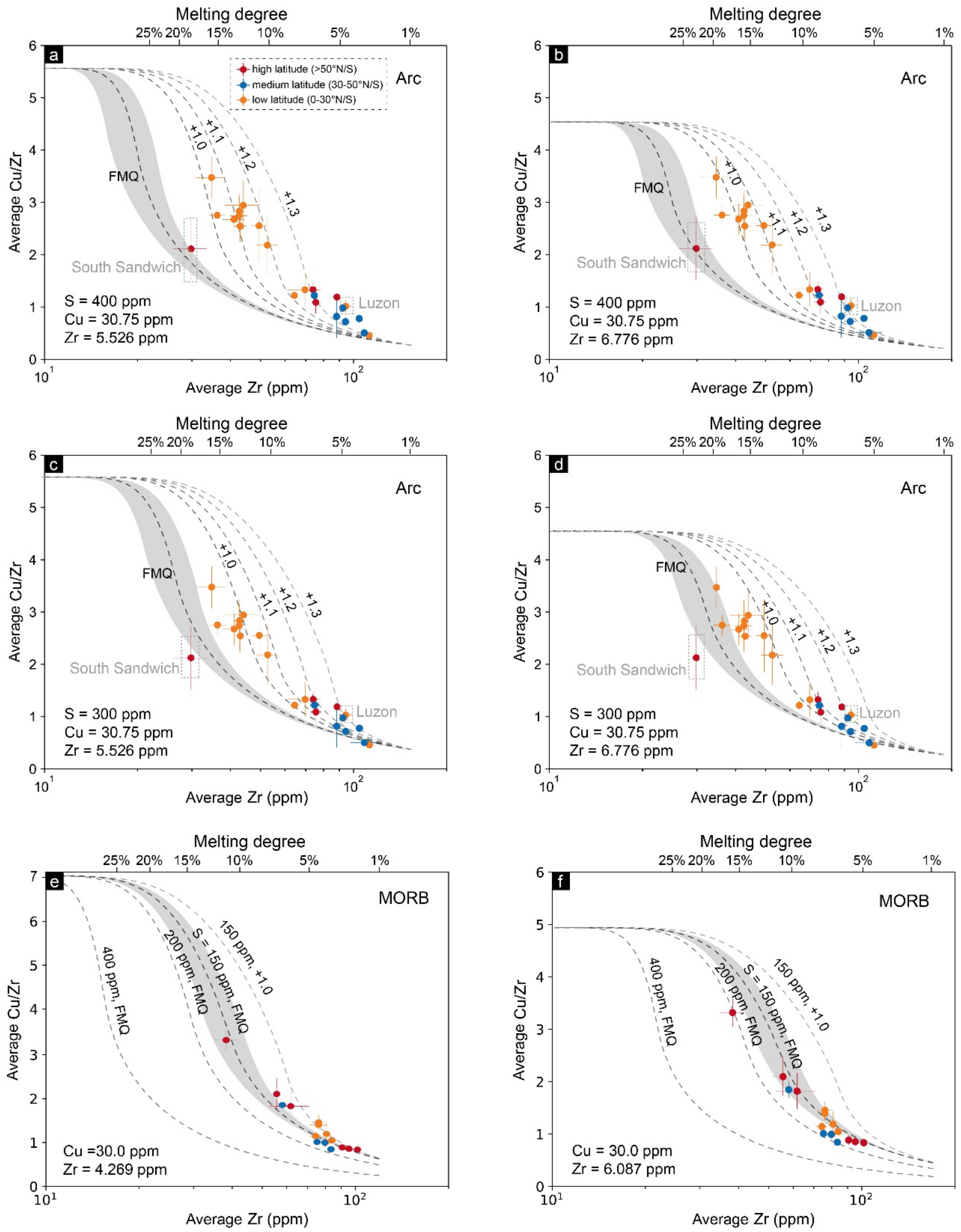

Supplementary Figure 6. Estimated oxygen fugacity of arc basaltic rocks and MORB based on Cu/Zr versus Zr (ppm). (a-d) The fO_2 estimation for arc basaltic rocks is modeled at S=400ppm or 300ppm, with Zr=5.526ppm or 6.776ppm for illustrating influence of source variations on oxygen fugacity estimation. (e, f) The fO_2 estimation for MORB is modeled with different mantle surfer contents at fO_2=FMQ (S=150ppm, 200ppm, and 400ppm), and at ΔFMQ=+1.0 (S=150ppm) with Zr=4.269ppm or 6.087ppm. The shaded regions represent 20% variations of sulfur solubility during mantle melting. The modeling results are listed in Supplementary Data 9 and 10. The error bar is 1SE.

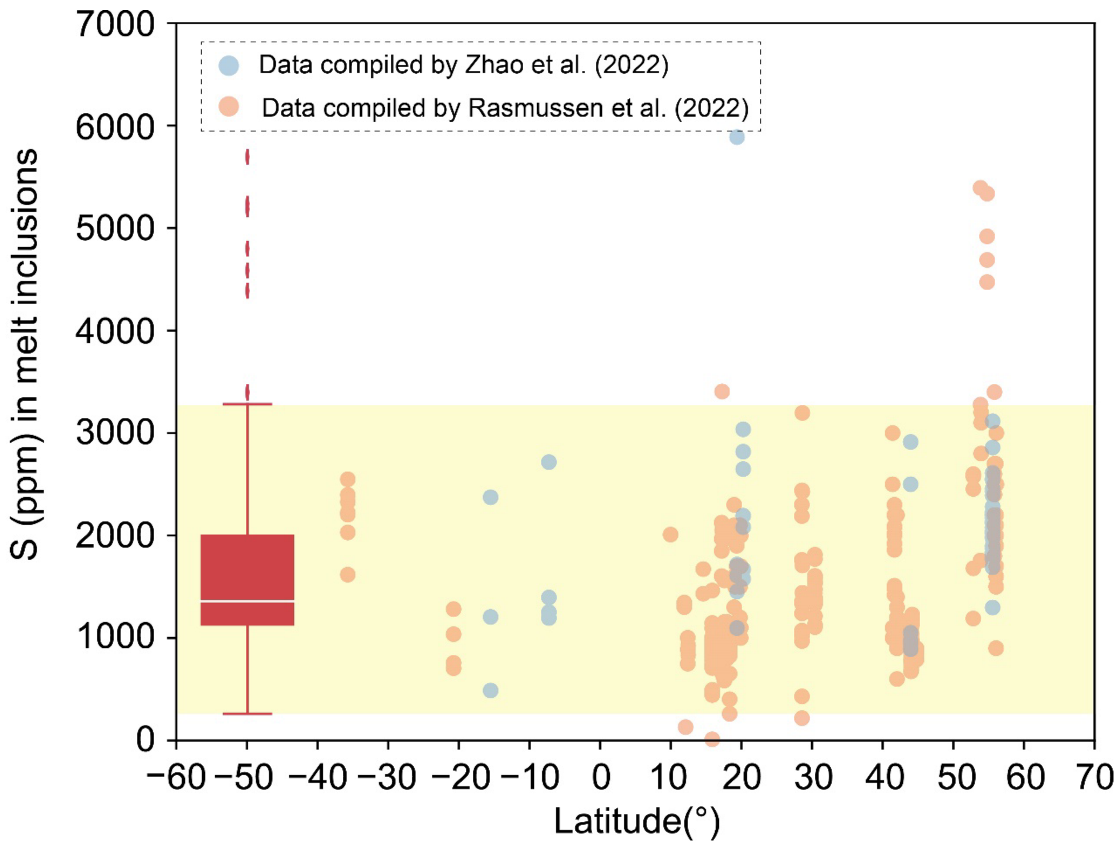

Supplementary Figure 7. Compiled data of sulfur content (ppm) in melt inclusions of volcanic rocks. The red box plot shows the analytical results of whole data with the majority data being between 1000 and 2000 ppm. The compiled data are listed in Supplementary Data 11. All the melt inclusion are from high Fo (>85) olivine with whole-rock Mg# higher than 60. No significant variations of sulfur contents in melt inclusion along the latitude.

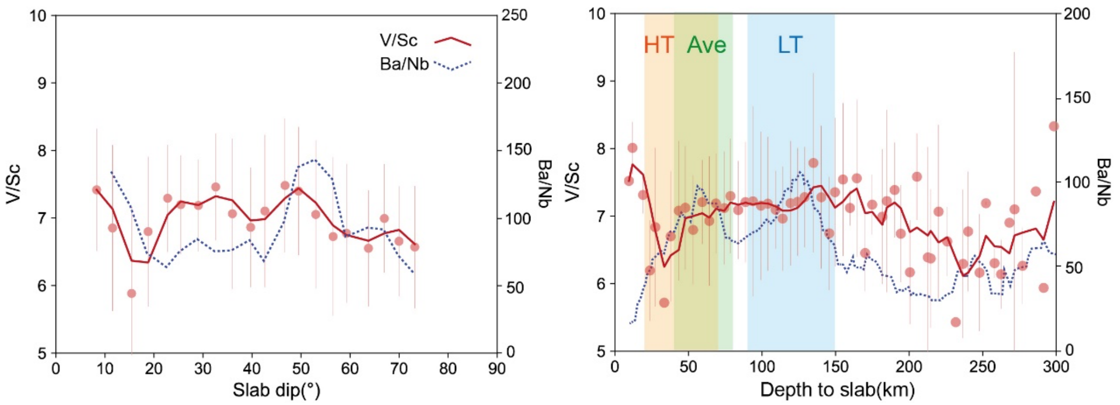

Supplementary Figure 8. Relationships between the slab thermal parameter, dehydration flux, and V/Sc ratios of primary arc basalts. (a) Relationships between the slab dip and V/Sc and Ba/Nb ratios of primary arc basalts. Red circles and thin lines represent average values and standard deviations of V/Sc ratios with a bin of 5°. Thick red line and blue dotted line represent calculated moving average curve of V/Sc and Ba/Nb ratios. No clear correlations between the V/Sc, Ba/Nb ratios and slab dip. The trends of V/Sc and Ba/Nb ratios vs. slab dip show some similarities, although the overall trends do not follow exactly. Compiled data are shown in Supplementary Data 2. (b) Relationships between the depth to slab and V/Sc and Ba/Nb ratios of primary arc basalts. Red circles and thin lines represent average values and standard deviations of V/Sc ratios with a bin of 5km. Thick red line and blue dotted line represent calculated moving average curve of V/Sc and Ba/Nb ratios. The Ba/Nb curve is according to Barber et al.[36]. HT, Ave, and LT represent the high temperature, average temperature, and low temperature H_2O flux condition in phase-equilibrium modeling, repectively[37].

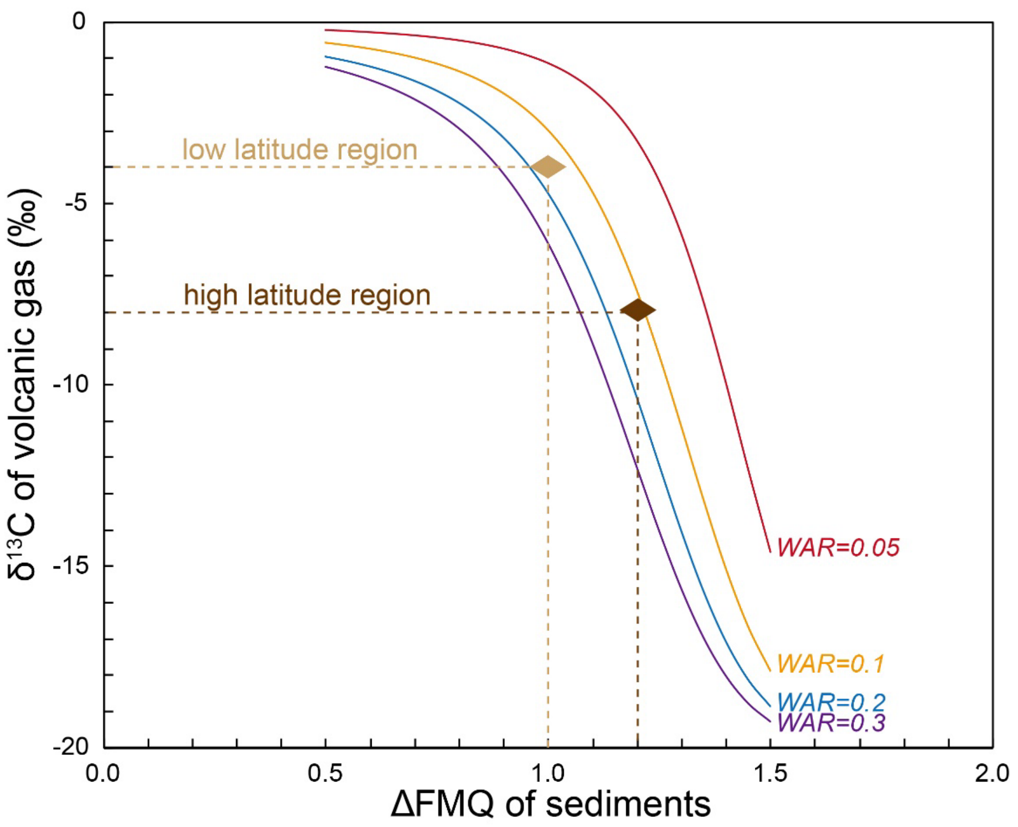

Supplementary Figure 9. Relationship between $\delta^{13}C$ of volcanic gases, ΔFMQ of sediments, and fluid-rock ratio. The equation used here is after Tumiati et al.[38]. WAR is the water-aragonite ratio and its range is followed Tumiati et al.[38]. The $\delta^{13}C$ of volcanic gases for low latitude region and high latitude region are based on the compilation from Mason et al.[39] (Supplementary Data 12). The calculated results show that the low latitude arcs with higher $\delta^{13}C$ of volcanic gases should have relatively lower ΔFMQ values of sediments.

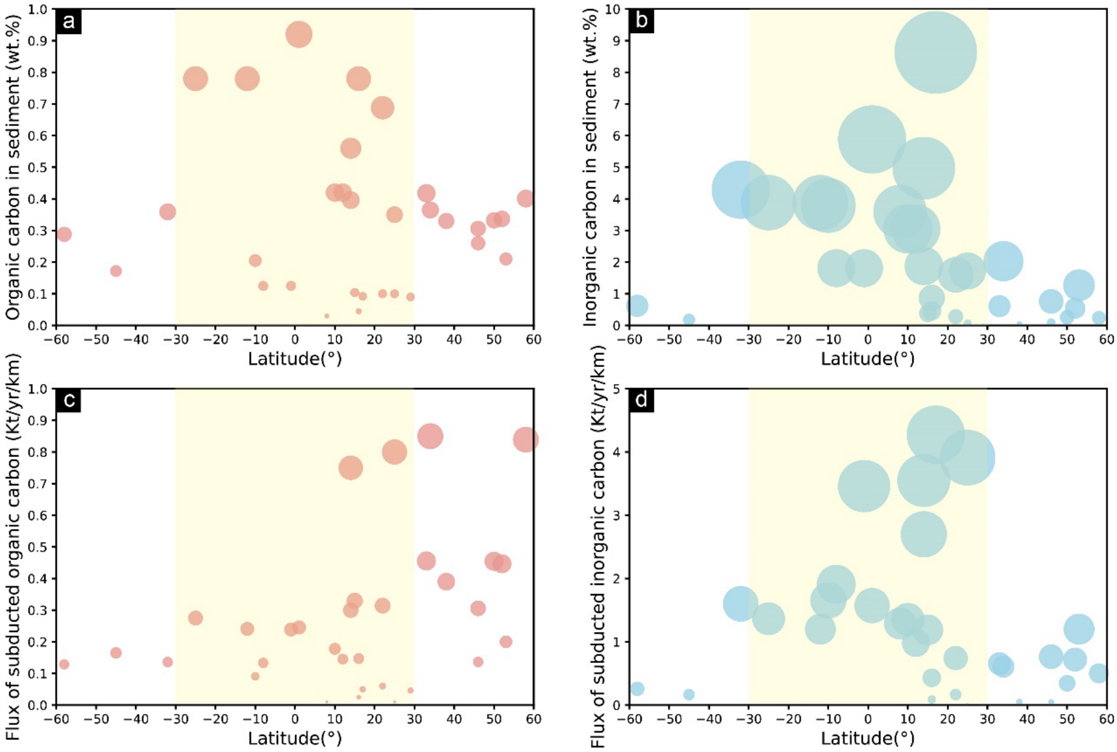

Supplementary Figure 10. Relationships between subducted carbon and latitude. (a, c) The contents (wt.%) and fluxes (Kt/yr/km) of organic carbon in trench sediments across the latitude. (b, d) The contents (wt.%) and fluxes (Kt/yr/km) of inorganic carbon in trench sediments across the latitude. The original data is from Clift[40]. The size of circles represents the values of contents and fluxes of organic or inorganic carbon in trench sediments. Compiled data are shown in Supplementary Data 14.

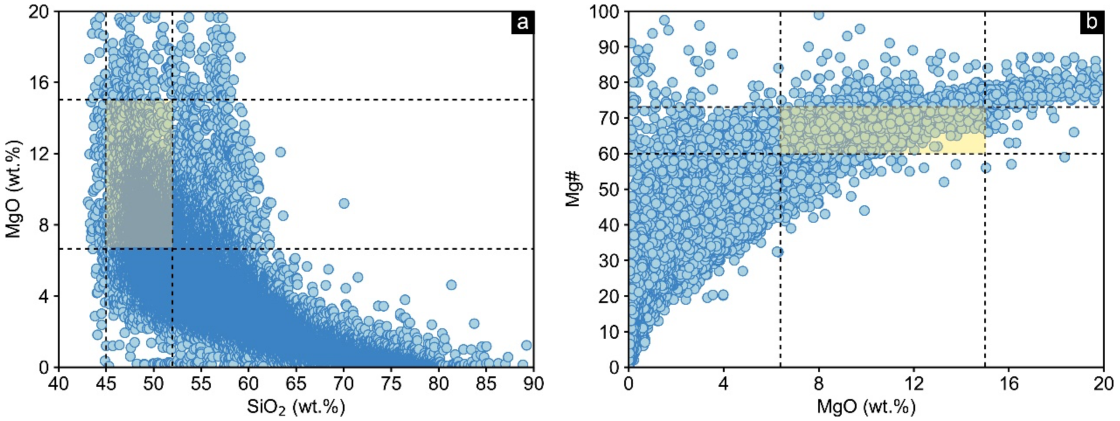

Supplementary Figure 11. Geochemical features and data filtration of compiled arc basaltic rocks. (a) MgO (wt.%) versus SiO_2 (wt.%). (b) Mg# (molar 100×MgO/(MgO+FeO_T))) versus MgO (wt.%). The dashed lines represent SiO_2, MgO, and Mg# filters.

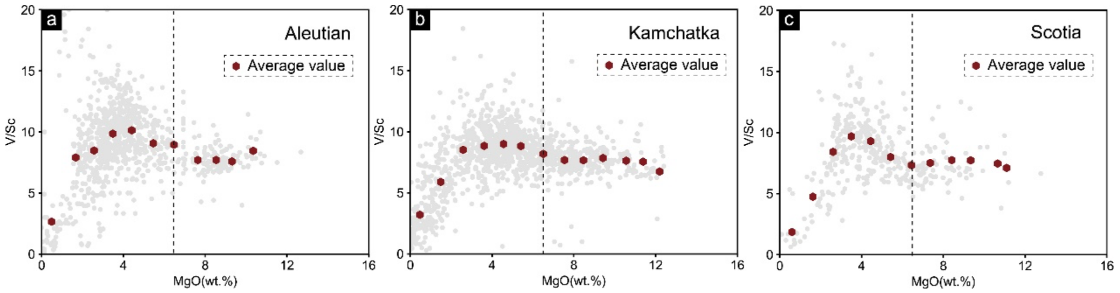

Supplementary Figure 12. V/Sc versus MgO (wt.%) of volcanic rocks in high latitude arcs. (a) The Aleutian arc. (b) The Kamchatka arc. (c) The Scotia arc. The grey circles are compiled data of each arc. The red hexagons represent average values of a 1 wt.% MgO bin. The plot shows the increasing of V/Sc ratios with decreasing MgO, typically when MgO < 6.5wt.%.

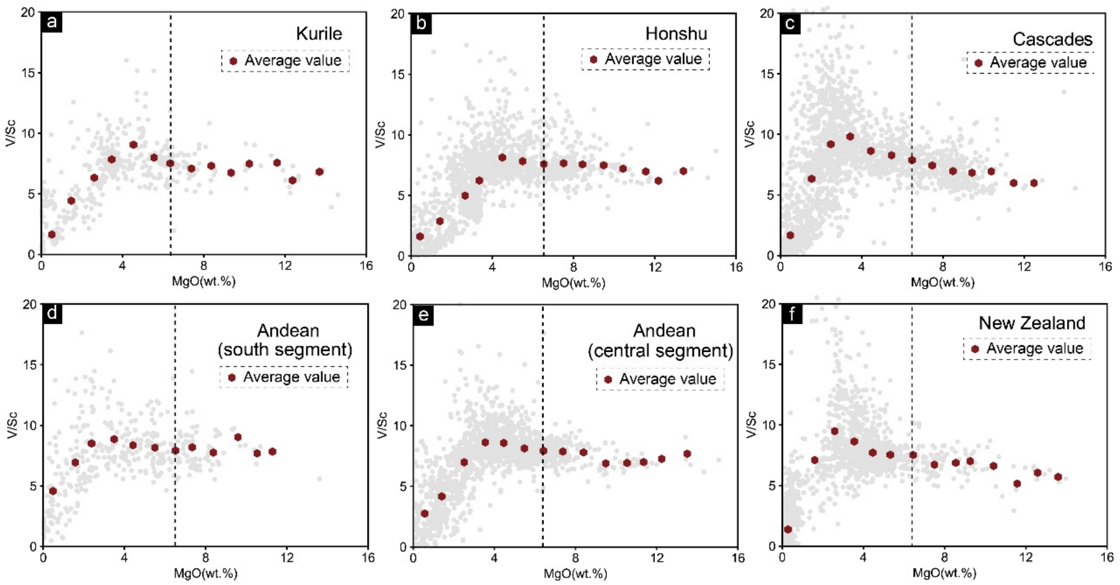

Supplementary Figure 13. V/Sc versus MgO (wt.%) of volcanic rocks in middle latitude arcs. (a) The Kurile arc. (b) The Honshu arc. (c) The Cascades arc. (d) The south segment of Andean arc. (e) The central segment of Andean arc. (f) The New Zealand arc. The grey circles are compiled data of each arc. The red hexagons represent average values of a 1 wt.% MgO bin. The plot shows the increasing of V/Sc ratios with decreasing MgO, typically when MgO < 6.5wt.%.

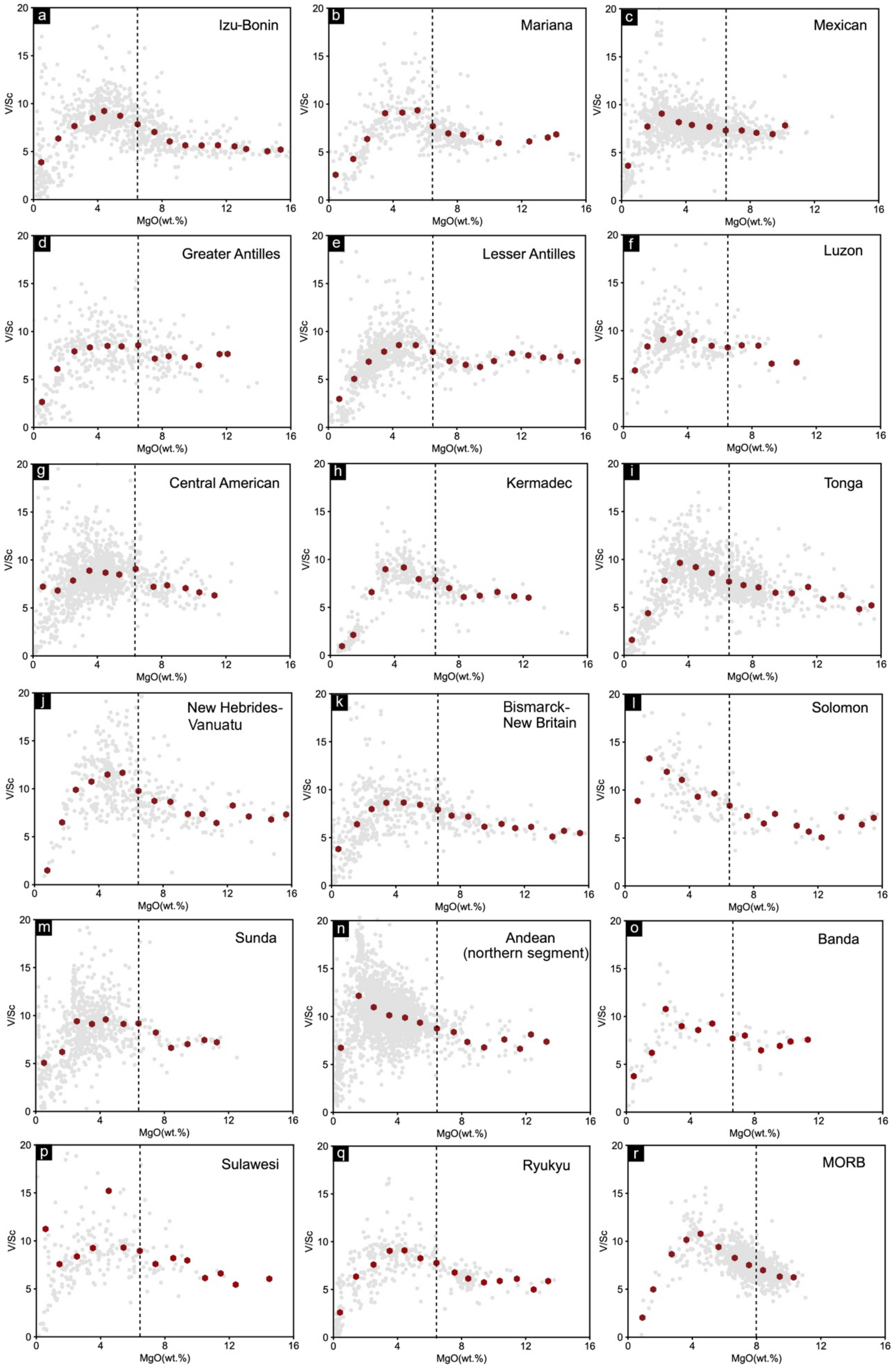

Supplementary Figure 14. V/Sc versus MgO (wt.%) of volcanic rocks in low latitude arcs and MORB. (a) The Izu-Bonin arc. (b) The Mariana arc. (c) The Mexican arc. (d) The Greater Antilles ac. (e) The Lesser Antilles arc. (f) The Luzon arc. (g) The Central American Volcanic Arc. (h) The

Kermadec arc. (i) The Tonga arc. (j) The New Hebrides-Vanuatu arc. (k) The Bismarck-New Britain arc. (l) The Solomon arc. (m) The Sunda arc. (n) The northern segment of Andean arc. (o) The Banda arc. (p) The Sulawesi arc. (q) The Ryukyu arc. (r) The global MORB data. The grey circles are compiled data of each arc or MORB. The red hexagons represent average values of a 1 wt.% MgO bin. The plot shows the increasing of V/Sc ratios with decreasing MgO, typically when MgO < 6.5wt.% for arc and 8.0% for MORB.

References:

1. Scotese, C. R. An Atlas of Phanerozoic Paleogeographic Maps: The Seas Come In and the Seas Go Out. *Annual Review of Earth and Planetary Sciences* **49**, 679–728 (2021).
2. Stolper, D. A. & Bucholz, C. E. Neoproterozoic to early Phanerozoic rise in island arc redox state due to deep ocean oxygenation and increased marine sulfate levels. *Proc. Natl. Acad. Sci. U.S.A.* **116**, 8746–8755 (2019).
3. Anser Li, Z.-X. & Aeolus Lee, C.-T. The constancy of upper mantle fO2 through time inferred from V/Sc ratios in basalts. *Earth and Planetary Science Letters* **228**, 483–493 (2004).
4. Aeolus Lee, C.-T., Leeman, W. P., Canil, D. & Li, Z.-X. A. Similar V/Sc Systematics in MORB and Arc Basalts: Implications for the Oxygen Fugacities of their Mantle Source Regions. *Journal of Petrology* **46**, 2313–2336 (2005).
5. Bucholz, C. E. & Kelemen, P. B. Oxygen fugacity at the base of the Talkeetna arc, Alaska. *Contrib Mineral Petrol* **174**, 79 (2019).
6. Wang, J. *et al.* Oxidation State of Arc Mantle Revealed by Partitioning of V, Sc, and Ti Between Mantle Minerals and Basaltic Melts. *J. Geophys. Res. Solid Earth* **124**, 4617–4638 (2019).
7. Turner, S. J. & Langmuir, C. H. A quantitative framework for global variations in arc geochemistry. *Earth and Planetary Science Letters* **584**, 117411 (2022).
8. Zhao, S.-Y., Yang, A. Y., Langmuir, C. H. & Zhao, T.-P. Oxidized primary arc magmas: Constraints from Cu/Zr systematics in global arc volcanics. *Sci. Adv.* **8**, eabk0718 (2022).

9. Turner, S. J. & Langmuir, C. H. The global chemical systematics of arc front stratovolcanoes: Evaluating the role of crustal processes. *Earth and Planetary Science Letters* **422**, 182–193 (2015).

10. Mallmann, G. & O'Neill, H. St. C. The Crystal/Melt Partitioning of V during Mantle Melting as a Function of Oxygen Fugacity Compared with some other Elements (Al, P, Ca, Sc, Ti, Cr, Fe, Ga, Y, Zr and Nb). *Journal of Petrology* **50**, 1765–1794 (2009).

11. Nakamura, E., Campbell, I. H., McCulloch, M. T. & Sun, S.-S. Chemical geodynamics in a back arc region around the Sea of Japan: Implications for the genesis of alkaline basalts in Japan, Korea, and China. *Journal of Geophysical Research: Solid Earth* **94**, 4634–4654 (1989).

12. Luhr, J. F., Allan, J. F., Carmichael, I. S. E., Nelson, S. A. & Hasenaka, T. Primitive calc-alkaline and alkaline rock types from the Western Mexican Volcanic Belt. *Journal of Geophysical Research: Solid Earth* **94**, 4515–4530 (1989).

13. Espinoza, F. *et al.* Petrogenesis of the Eocene and Mio–Pliocene alkaline basaltic magmatism in Meseta Chile Chico, southern Patagonia, Chile: Evidence for the participation of two slab windows. *Lithos* **82**, 315–343 (2005).

14. Mantle, G. W. & Collins, W. J. Quantifying crustal thickness variations in evolving orogens: Correlation between arc basalt composition and Moho depth. *Geology* **36**, 87–90 (2008).

15. Cameron, W. E., McCulloch, M. T. & Walker, D. A. Boninite petrogenesis: Chemical and Nd-Sr isotopic constraints. *Earth and Planetary Science Letters* **65**, 75–89 (1983).

16. Crawford, A. J. Classification, petrogenesis and tectonic setting of boninites. *Boninites and Related Rocks* (1989).

17. Falloon, T. J. & Danyushevsky, L. V. Melting of Refractory Mantle at 1·5, 2 and 2·5 GPa under

Anhydrous and H2O-undersaturated Conditions: Implications for the Petrogenesis of High-Ca Boninites and the Influence of Subduction Components on Mantle Melting. *Journal of Petrology* **41**, 257–283 (2000).

18. Falloon, T. J. *et al.* Boninites and Adakites from the Northern Termination of the Tonga Trench: Implications for Adakite Petrogenesis. *Journal of Petrology* **49**, 697–715 (2008).
19. Kelemen, P. B., Hanghøj, K. & Greene, A. R. One View of the Geochemistry of Subduction-Related Magmatic Arcs, with an Emphasis on Primitive Andesite and Lower Crust. in (eds. Holland, H. D. & Turekian, K. K.) vol. 4 749–806 (Elsevier, Amsterdam, 2014).
20. Defant, M. J. & Drummond, M. S. Derivation of some modern arc magmas by melting of young subducted lithosphere. *Nature* **347**, 662–665 (1990).
21. Yang, X.-M. Using the Rittmann Serial Index to define the alkalinity of igneous rocks. *Neues Jahrbuch für Mineralogie - Abhandlungen* **184**, 95–103 (2007).
22. Raos, A. M. & Crawford, A. J. Basalts from the Efate Island Group, central section of the Vanuatu arc, SW Pacific: geochemistry and petrogenesis. *Journal of Volcanology and Geothermal Research* **134**, 35–56 (2004).
23. Gazel, E. *et al.* Galapagos-OIB signature in southern Central America: Mantle refertilization by arc–hot spot interaction. *Geochemistry, Geophysics, Geosystems* **10**, (2009).
24. Hoernle, K. *et al.* Arc-parallel flow in the mantle wedge beneath Costa Rica and Nicaragua. *Nature* **451**, 1094–1097 (2008).
25. Tonarini, S., Leeman, W. P. & Leat, P. T. Subduction erosion of forearc mantle wedge implicated in the genesis of the South Sandwich Island (SSI) arc: Evidence from boron isotope systematics. *Earth and Planetary Science Letters* **301**, 275–284 (2011).

26. Zhang, Y., Gazel, E., Gaetani, G. A. & Klein, F. Serpentinite-derived slab fluids control the oxidation state of the subarc mantle. *Sci. Adv.* **7**, eabj2515 (2021).

27. Plank, T. & Manning, C. E. Subducting carbon. *Nature* **574**, 343–352 (2019).

28. Haase, K. M., Beier, C., Fretzdorff, S., Smellie, J. L. & Garbe-Schönberg, D. Magmatic evolution of the South Shetland Islands, Antarctica, and implications for continental crust formation. *Contrib Mineral Petrol* **163**, 1103–1119 (2012).

29. Anderson, D. W. *et al.* Tracing mantle components and the effect of subduction processes beneath the northern Antarctic Peninsula. *Geochimica et Cosmochimica Acta* **343**, 234–249 (2023).

30. Gao, L. *et al.* Oxidation of Archean upper mantle caused by crustal recycling. *Nat Commun* **13**, 3283 (2022).

31. Salters, V. J. M. & Stracke, A. Composition of the depleted mantle. *Geochemistry, Geophysics, Geosystems* **5**, (2004).

32. Nicklas, R. W. *et al.* Secular mantle oxidation across the Archean-Proterozoic boundary: Evidence from V partitioning in komatiites and picrites. *Geochimica et Cosmochimica Acta* **250**, 49–75 (2019).

33. Aulbach, S. & Stagno, V. Evidence for a reducing Archean ambient mantle and its effects on the carbon cycle. *Geology* **44**, 751–754 (2016).

34. Brounce, M. N. A geochemical investigation of oxygen fugacity in the Marianas subduction factory. (University of Rhode Island, 2014).

35. Lee, C.-T. A., Luffi, P., Plank, T., Dalton, H. & Leeman, W. P. Constraints on the depths and temperatures of basaltic magma generation on Earth and other terrestrial planets using new

thermobarometers for mafic magmas. *Earth and Planetary Science Letters* **279**, 20–33 (2009).

36. Barber, N. D., Edmonds, M., Jenner, F. & Williams, H. Global Ba/Nb systematics in arc magmas reflect the depths of mineral dehydration in subducted slabs. *Geology* **50**, 1438–1442 (2022).

37. Hernández-Uribe, D. & Palin, R. M. A revised petrological model for subducted oceanic crust: Insights from phase equilibrium modelling. *Journal of Metamorphic Geology* **37**, 745–768 (2019).

38. Tumiati, S. *et al.* Subducted organic matter buffered by marine carbonate rules the carbon isotopic signature of arc emissions. *Nat Commun* **13**, 2909 (2022).

39. Mason, E., Edmonds, M. & Turchyn, A. V. Remobilization of crustal carbon may dominate volcanic arc emissions. *Science* **357**, 290–294 (2017).

40. Clift, P. D. A revised budget for Cenozoic sedimentary carbon subduction: Cenozoic Carbon Subduction. *Rev. Geophys.* **55**, 97–125 (2017).
