# Supplementary material for: Latitude-dependent oxygen fugacity in arc magmas
Source: Nat Commun. 2024 Jul 18;15:6050. doi: 10.1038/s41467-024-50337-6 (PMC11258285; doi:10.1038/s41467-024-50337-6)
Supplement: Supplementary file 3 — Description of Additional Supplementary Files [file 41467_2024_50337_MOESM3_ESM.pdf]

Description of Additional Supplementary Files

Supplementary Data 1. Compiled measured oxygen fucacity data (ΔFMQ) of basaltic rocks in different arc segments.

Supplementary Data 2. Compiled slab's physical properties and geochemical data of Cenozoic primary basaltic rocks in different arc segments.

Supplementary Data 3. Compiled data of burial flux of carbonate on seafloor in neritic zone.

Supplementary Data 4. Compiled geochemical data of global mid-ocecan ridge basalt.

Supplementary Data 5. Summarized average values of slab's physical properties and geochemical data of primary basaltic rocks in different arc segments.

Supplementary Data 6. Parameters used for Sr-Nd isotope mixing modeling.

Supplementary Data 7. Model for V, Sc and Ti variations during sub-arc mantle melting.

Supplementary Data 8. Model for V, Sc and Ti variations during mid-ocean ridge mantle melting.

Supplementary Data 9. Model for Cu and Zr variations during sub-arc mantle melting.

Supplementary Data 10. Model for Cu and Zr variations during mid-ocean ridge mantle melting.

Supplementary Data 11. Summerized sulfur content in olivine inclusions from primary basaltic rocks.

Supplementary Data 12. Compiled data of $\delta^{13}C$ of volcanic arc gases.

Supplementary Data 13. Compiled data of particulate organic carbon (POC) flux at 2000m.

Supplementary Data 14. Compiled data of total carbon in subducted sediments and flux of subducted total carbon.

Supplementary Data 15. Compiled data of sulfur isotope of melt inclusions in volcanic rocks and harzburgite, magmatic sulfides in cumulate, and whole-rock samples from different arc segments.
